# Bioterrorism-Related Anthrax Surveillance, Connecticut, September–December, 2001

**DOI:** 10.3201/eid0810.020399

**Published:** 2002-10

**Authors:** Alcia A. Williams, Umesh D. Parashar, Adrian Stoica, Renee Ridzon, David L. Kirschke, Richard F. Meyer, Jennifer McClellan, Marc Fischer, Randy Nelson, Matt Cartter, James L. Hadler, John A. Jernigan, Eric E. Mast, David L. Swerdlow, the Connecticut Anthrax Investigation Team

**Affiliations:** * Centers for Disease Control and Prevention, Atlanta, Georgia, USA; † Connecticut State Department of Public Health, Hartford, Connecticut, USA

## Abstract

On November 19, 2001, a case of inhalational anthrax was identified in a 94-year-old Connecticut woman, who later died. We conducted intensive surveillance for additional anthrax cases, which included collecting data from hospitals, emergency departments, private practitioners, death certificates, postal facilities, veterinarians, and the state medical examiner. No additional cases of anthrax were identified. The absence of additional anthrax cases argued against an intentional environmental release of *Bacillus anthracis* in Connecticut and suggested that, if the source of anthrax had been cross-contaminated mail, the risk for anthrax in this setting was very low**.** This surveillance system provides a model that can be adapted for use in similar emergency settings.

In response to the World Trade Center attack on September 11, 2001, the Connecticut Department of Public Health, assisted by all Connecticut hospitals, implemented a syndromic surveillance system that monitored admissions to acute-care hospitals and visits to emergency departments to detect any concurrent bioterrorism event. All hospitals and emergency departments were asked to report six categories of admissions: respiratory conditions of any type, pneumonia, meningitis or encephalitis, paralysis or paresis of nontraumatic origin, clusters of unusual illness, and total admissions on a daily basis.

After the first confirmed inhalational anthrax case on October 4 (1), the surveillance system was modified to detect the early phase of any disease outbreak that might occur as a result of mass exposure to biological agents—bacteria, viruses, or toxins—used for terrorism. Seven additional hospital admission categories were included in the surveillance system: hemoptysis, acute respiratory distress syndrome or respiratory failure of uncertain origin, sepsis or nontraumatic shock, fever and rash, fever of unknown origin, gastrointestinal symptoms (vomiting, diarrhea, and dehydration), and skin infections.

On November 20, 2001, the 11th known case of bioterrorism-related inhalational anthrax since October 4 was identified in a 94-year-old resident of Oxford, Connecticut, a rural community of <10,000 persons. Unlike most recent patients with bioterrorism-associated anthrax, this patient was not a media or postal worker (1–4). A team of public health investigators from the Centers of Disease Control and Prevention was invited by the state of Connecticut to work in collaboration with state and local health officials to conduct an epidemiologic investigation.

After the death of the index patient on November 21, ongoing statewide surveillance for bioterrorism-related disease was expanded to meet two objectives: 1) conduct retrospective surveillance to identify any previously undetected cases of anthrax since September 1, 2001, that might provide clues to the source of exposure and to assess the possibility of intentional environmental release of *Bacillus anthracis* and 2) conduct prospective surveillance to detect early cases of anthrax that might occur and ensure rapid detection and treatment. The surveillance activities that occurred during this epidemiologic investigation are described.

## Methods

### Surveillance Activities

Because the first identified case of bioterrorism-related human anthrax in the United States (1) had a presumed source of exposure in mid-September 2001, retrospective surveillance focused on the period from September 1 to November 30, 2001. Methods included reviewing death certificates, laboratory data, medical examiner’s records, and postal worker absentee records to find evidence of illness in the general population and conducting a veterinary survey to seek evidence in animal populations. Prospective surveillance focused on the period beginning November 21, 2001, and included hospital admissions, emergency department visits, and private physician reports. After retrospective surveillance was completed, we also initiated prospective surveillance of medical examiner and postal worker absentee records.

### Case Definitions

We defined a confirmed case of anthrax as clinically compatible illness in a person with laboratory confirmation by isolation of *B. anthracis* from a clinical specimen or other laboratory evidence of *B. anthracis* infection based on at least two supportive laboratory tests (e.g., polymerase chain reaction or serologic or immunohistochemical testing). We defined a suspected case as a clinically compatible case of illness without isolation of *B. anthracis* and no alternative diagnosis, but with laboratory evidence of *B. anthracis* by one supportive laboratory test; or a clinically compatible case of anthrax linked by epidemiologic methods to a confirmed environmental exposure but without corroborative laboratory evidence of infection. Illnesses that were investigated and failed to fulfill criteria for the above case definitions were classified as “no apparent anthrax disease.”

## Retrospective Surveillance

### Death Certificates

All death certificates for persons who died in Connecticut from September 1 to November 30 were reviewed to ascertain if any deaths could be potentially associated with anthrax. Because of the central role of contaminated letters in previous anthrax cases, surveillance focused on deaths occurring in Oxford, where the patient lived, and the eight surrounding towns (Ansonia, Beacon Falls, Derby, Naugatuck, Seymour, Shelton, Southbury, and Woodbury [total population 152,481]) served by the same postal processing and distribution center in Wallingford, Connecticut. This facility received mail from postal distribution facilities known to be contaminated by *B. anthracis* spores, including the postal center in Hamilton, New Jersey, where the envelopes containing *B. anthracis* sent to two U.S. senators originated.

Death certificates with the following conditions listed as the immediate or underlying cause of death were selected for further review: pneumonia, sepsis, cardiac arrest without cause, respiratory arrest without cause, sudden death, and undetermined cause. Deaths were further classified by place of occurrence: hospital, nursing home, residence, or other setting. Because of the paucity of clinical information on deaths occurring outside hospitals, the review focused on in-hospital deaths.

To obtain additional information on in-hospital deaths, laboratories, infection control practitioners, and physicians were contacted by telephone to identify patients for whom a definitive cause of death could be determined. For the remaining deaths in which cause of death could not be ascertained, medical record reviews by a team of four physician epidemiologists using a standardized abstraction form were conducted at the hospitals where the deaths occurred.

### Laboratory Data

Hospital-associated laboratories statewide were contacted to obtain information on any gram-positive rods or *Bacillus* species isolated from sterile sites (e.g., blood, cerebrospinal fluid, or pleural fluid). A standardized reporting form was provided to laboratories to be completed and sent to a 24-hour–accessible fax machine. For *Bacillus* species isolates, we contacted laboratories by phone to gather information about motility and hemolysis tests when this information was not provided on the report. For all other reports of gram-positive bacilli, laboratories were contacted to obtain speciation information if available, when this information was not provided. All available isolates suspicious for *B. anthracis* were sent to the Connecticut Department of Public Health (CDPH) laboratory for final identification.

### Medical Examiner’s Records

Connecticut’s state medical examiner is notified of deaths that occur outside hospitals or within 24 hours of hospitalization. Data on deaths referred to the medical examiner and reported from September 1 to November 26 were reviewed. After November 26, ongoing prospective surveillance for deaths referred to the medical examiner was assumed by CDPH, with a particular focus on deaths in the town where the index patient resided and the eight surrounding towns. The medical examiner’s office and CDPH made the decision about whether an autopsy was necessary to exclude anthrax as the cause of death, based on the symptoms of the deceased patient and the clinical circumstances surrounding death.

### Postal Worker Absenteeism

Work attendance records were obtained from both the local postal and main processing distribution facilities serving the index patient’s town of residence and the eight surrounding towns (Seymour and Wallingford postal facilities). To obtain information about reasons for absence, either postal management or CDPH personnel interviewed postal workers with absences for >3 consecutive days from September 11 to November 25, 2001. When workers were not available to be interviewed, information was obtained by interviewing management personnel, who also were questioned about recent deaths in postal workers.

### Surveillance for Postal Worker Influenzalike Illness and Cutaneous Conditions

The U.S. Postal Service had been conducting surveillance for influenzalike illness or cutaneous conditions compatible with anthrax among postal workers nationwide since October 25, 2001. In Connecticut, postal service management collected data from postal workers and reported to the postal medical office in Hartford, Connecticut. Reports from employees were voluntary. Data for the state were submitted to area headquarters (serving New England and parts of New York) daily and then reported to national postal headquarters in Washington, D.C. For cases in which the postal worker was hospitalized with influenzalike symptoms, national headquarters was notified directly. Beginning November 6, only hospitalizations were reported to area headquarters; however, data for the state of Connecticut were still collected in Hartford. All past reports to the system and ongoing reports were reviewed to characterize the symptoms and signs; for conditions suspicious for anthrax, health-care providers were called for further clinical information.

## Prospective Surveillance

### Hospital, Emergency Departments, and Physician Reports

The statewide hospital-based surveillance for bioterrorism-related agents that began after September 11, 2001, was enhanced from November 27 to December 15. All acute-care hospitals in Connecticut designated a surveillance officer (e.g., infection control practitioner, nurse, or physician) who would be responsible for surveillance of conditions potentially related to anthrax and other bioterrorism-related agents at their institution. Each day, the surveillance officer contacted the clinical microbiology laboratory to request a list of any suspect Gram stain results or bacterial isolates from sterile sites. Suspect results were defined as gram-positive rods that had not been further identified or *Bacillus* species that had not been further typed or for which speciation as *B. anthracis* had not been excluded. Additionally, the surveillance officer reviewed admissions for the previous 24 h and reported patients having any one of five clinical syndromes (acute respiratory failure with pleural effusion; hemorrhagic enteritis with fever; a skin lesion characterized by vesicles, ulcer, or eschar; meningitis, encephalitis, or unexplained acute encephalopathy; or anthrax or suspected anthrax infection) and a widened mediastinum on chest radiograph or laboratory findings of a gram-positive bacillus on Gram stain, *Bacillus* species from culture of a sterile site specimen, or hemorrhagic cerebrospinal fluid, pleural, or peritoneal fluid in patients without a traumatic tap or event.

Using a standardized form, the surveillance officer reported findings daily to CDPH. Upon identifying patients with the surveillance criteria for a suspect anthrax case, hospital surveillance officers contacted a designated member of the surveillance team by telephone and faxed the report. These patients were then referred to a clinical team for further evaluation. In addition, physicians and infection control practitioners statewide (in particular those in the nine towns including and surrounding the town of the index patient) were asked to report immediately to CDPH any patient with symptoms that suggested anthrax.

## Other Anthrax Surveillance Activities

### Survey of Veterinary Practices

To ascertain undiagnosed animal anthrax cases, a one-page questionnaire was distributed to the members of the Connecticut Veterinary Medical Association (CVMA) on November 28. CVMA has a total of 620 members, accounting for 82% of the 768 CDPH-licensed veterinarians in Connecticut. Information collected included the number of veterinarians associated with the practice, type of practice, number of undiagnosed deaths by animal species, animal deaths accompanied by clinical signs consistent with anthrax, and knowledge of confirmed cases of animal anthrax in Connecticut. Questionnaires were sent by the CVMA rapid fax system to the approximately 325 members who requested faxed updates from CDPH. We requested a single completed questionnaire from each practice. Since some practices included veterinarians who are not CVMA members, the survey likely reached more veterinarians than actual members who had requested faxed updates.

## Results

Data were entered and analyzed in an Epi Info database (5). Hospital, emergency department, and physician reports were evaluated at least twice a day.

Among the 487 deaths reported from the nine towns in September, October, and November 2001, a total of 131 (26.9 %) had one of the six conditions under surveillance. Of these, 66 (50.3%) occurred in hospitals; the rest occurred in residences, nursing homes, and other settings. No postmortem examinations were performed. By contacting physicians, infection control practitioners, and laboratories, a likely cause of death other than anthrax was identified for 7 (10.6%) patients. For the remaining 59 (89.4%) patients, medical record review was necessary. In 33 (55.9%), a cause of death other than anthrax was identified. For 12 (20.3%) patients, the cause of death was not apparent, but available information on the clinical features and clinical course (such as absence of fever and respiratory symptoms) of the patients did not suggest a diagnosis of anthrax. Insufficient data were available to assess the cause of death for 14 (23.7%) patients because death occurred before or shortly after arrival to the hospital. None of these patients had been autopsied, and because of the lack of a clear indication and the limited availability of resources, no further measures (e.g., exhuming the body to conduct autopsy) were taken to ascertain the cause of death.

### Laboratory Data

Thirty (96.7%) of 31 clinical laboratories provided data. Twenty-two (73.3%) laboratories reported at least one patient with a gram-positive bacillus or *Bacillus* species isolate. Gram-positive bacilli were identified in 71 specimens from 70 patients (one patient had more than one specimen submitted), including blood (59 specimens), tissue (6 specimens), peritoneal fluid (3 specimens), pleural fluid (2 specimens), and 1 surgical site specimen. Of patients with gram-positive bacilli, 49 had *Bacillus* species isolated; none of these was identified as *B. anthracis*. For the remaining 22 reports of gram-positive bacilli, 16 were identified as *Corynebacterium*, 1 as *Proprionobacterium*, 1 as *Clostridium*, 1 as *Eubacterium*, and 1 as *Staphylococcus hominis*; 1 was a mixed infection with gram-positive organisms, and 1 was an unidentified motile gram-variable bacillus.

### Medical Examiner’s Records

One hundred forty-eight deaths were reported to the medical examiner. Of these, autopsies were performed on 14 (9.4%) patients. Cause of death was determined to be an accident in six, cardiac disease in four, suicide in three, and inhalational anthrax in one (the index patient). Because of the lack of clinical information on the remaining patients who had not been autopsied, further review was not possible.

### Postal Worker Absenteeism

At the local postal facility in Seymour, no employees died during the surveillance period. Two persons were absent for >3 days, one for a scheduled surgery and the other for an injury. At the main processing and distribution center in Wallingford, two recent postal worker deaths were attributed to cardiovascular disease; both occurred before September 11, 2001. Approximately 35 employees were absent for >3 consecutive days. Interviews of the postal workers about the reasons for absence showed no apparent anthrax in any workers.

### Postal Worker Influenzalike Illness and Cutaneous Lesion Surveillance

Ninety-two reports of influenzalike illness were reviewed. For seven patients with characteristics that might have been compatible with anthrax (e.g., cutaneous lesions, influenzalike illness with absence of rhinorrhea, and shortness of breath), further clinical information was obtained. All cases were classified as “no apparent anthrax disease” after review.

## Prospective Surveillance

### Hospital, Emergency Departments, and Physician Reports

Of 59 reports received, all were classified as “no apparent anthrax disease.” Specimens from 14 patients were sent to CDC, including 15 serum specimens, 14 skin biopsy specimens, 3 lung biopsies, 2 samples of pleural fluid, 11 samples of whole blood for polymerase chain reaction, and (from one patient) autopsy specimens from the gastrointestinal tract, liver, a lymph node, and one mixed tissue specimen.

## Other Anthrax Surveillance Activities

### Survey of Veterinary Practices

A total of 140 questionnaires were returned from 140 practices, representing 365 veterinarians and 48% of licensed Connecticut veterinarians. Completed questionnaires were received from practices distributed throughout eight counties of the state. Of these, 113 (81%) were small animal practices; 14 (10%) a mixture of small animals, equine, and food animal practices; and 12 (9%) equine practices. Of the respondents, 69 practices with 180 veterinarians, including nine practices and 20 livestock veterinarians, were located in the two counties representing the nine towns of interest during surveillance. Of the 140 practices, 18 (13%) reported that they were aware of undiagnosed animal deaths since September 15, 2001. None of the respondents indicated that they or anyone in their practice knew of a confirmed case of animal anthrax in Connecticut.

## Discussion

Despite intensive, active prospective and retrospective surveillance, we did not identify any patients other than the index case with features compatible with anthrax. This finding indicates that the index patient was probably not exposed through intentional local environmental release of *B. anthracis;* therefore, the concurrent epidemiologic investigation focused on the personal activities and contacts of the patient. Our findings, in conjunction with the *B. anthracis* contamination of the regional postal distribution facility, suggest that the index patient was likely exposed through cross-contaminated mail. If so, the lack of additional anthrax cases among persons who received mail from the same postal facility as the index patient also suggests that the risk from cross-contaminated mail in this setting was very low.

The scope of this epidemiologic investigation did not include a formal evaluation of the surveillance system. Although a standard for evaluating the performance of a system to detect covert acts of bioterrorism has not yet been described, we have some general comments about the traditional criteria (6) used in assessing the attributes of surveillance systems. Our system was complex and labor-intensive, requiring an estimated 1,500 person-hours for state and federal public health officials alone during the most intense 3-week period of the investigation. However, the system operated effectively. The acceptance of the system and compliance in reporting were likely enhanced by both national and local events—the World Trade Center and Pentagon attacks, the subsequent anthrax–tainted mailings, and the death of the Connecticut resident from anthrax. The staff at the public health department were highly motivated, and training requirements were minimal because of their knowledge of the preexisting system for syndromic surveillance. Use of existing resources provided a foundation for successfully implementing enhanced surveillance in less than 12 hours. Because of standardized and relatively simple reporting forms and data abstraction by trained investigators, quality of the data was excellent. The system was by design flexible and met evolving needs, including adding new syndromes to the surveillance system and moving staff from one activity to another as needed. Centralized reporting by fax or telephone assisted us in identifying early any problems in implementation of the surveillance system.

The true frequency of reportable syndromes was not known before we implemented this surveillance system for bioterrorism-related agents. Furthermore, with no prior knowledge of bioterrorism events, adequate numerator for the occurrence of any bioterrorism-related syndrome, or denominator for the population susceptible to the event, calculating the sensitivity and predictive value positive for the system was difficult. However, this system likely reflected accurately the lack of additional anthrax cases in both animal and human populations in Connecticut. Approximately 80% of Connecticut-licensed veterinarians in the state were successfully surveyed, including veterinarians who treat livestock most susceptible to anthrax infection, and none reported any animal illness consistent with anthrax. Similarly, an exhaustive search for human anthrax cases based on review of clinical and laboratory data yielded no additional cases.

In general, we received timely data that ensured quick and appropriate public health responses and allowed modifications to the system as needed. For hospital reporting, most reports were transmitted to a designated fax by noon each day for events during the preceding day. This plan was not problematic except on weekends, when hospitals were often operating with minimal staff. Without exception, all hospitals submitted data no later than 4 p.m. on the day of required reporting. Frequently, hospital, laboratory, medical examiner, and postal service personnel contacted a member of the team by telephone or pager with concerns about potential patients with suspect symptoms. The Connecticut Vital Records Department directed the daily transmission of all death certificates from the towns of interest, which allowed for continual monitoring of suspected deaths that required further investigation. The surveillance system operated 24 hours a day, 7 days a week; surveillance team members were always available. This constant accessibility was helpful with data turnaround and evaluation of suspect cases but difficult to sustain and resource intensive. Although surveillance instruments evolved over time, these changes did not detract from the ability to collect, manage, and disseminate the data, attesting to the stability of the system.

Our surveillance activities met the objectives of providing information about the source of exposure for the index case and guiding the course of the accompanying epidemiologic investigation. Although we were able to approach “real-time” reporting, permanent sustainability of these activities is unrealistic because they require too many resources. While the costs of sustaining this system were not directly evaluated, such an analysis would be useful. Explicit discussion of costs and benefits may help both in terms of protecting and increasing funding levels and assuring that existing surveillance systems are necessary and make the best possible use of limited resources. In situations requiring surveillance, an approach similar to ours could be applied after suitable modifications to meet the need for short periods of time. Clearly, the approaches to detecting sentinel bioterrorism events require further evaluation, standardization, and improvements to allow a timely, efficient, and effective public health response.
